# An innovative Indigenous-led model for integrated COVID-19 case management in Auckland, New Zealand: lessons from implementation

**DOI:** 10.3389/fpubh.2024.1324239

**Published:** 2024-02-09

**Authors:** Elana Curtis, Belinda Loring, Kadin Latham, Anthony Jordan, Nigel Chee, Rangimarie Hunia, Karl Snowden, Kerry Tari, Paora Murupaenga, Roimata Tipene, Stevie Whitcombe, Kelleigh Embers, Rawiri McKree Jansen

**Affiliations:** ^1^Te Kupenga Hauora Māori, Faculty of Medical and Health Sciences, University of Auckland, Auckland, New Zealand; ^2^Te Aka Whai Ora – Māori Health Authority, Auckland, New Zealand; ^3^Te Whatu Ora - Te Toka Tumai Auckland, Auckland, New Zealand; ^4^Ngāti Whātua Ōrakei Whai Maia, Auckland, New Zealand; ^5^Autonomy Health, Auckland, New Zealand; ^6^Te Whatu Ora – Health New Zealand Counties Manukau, Auckland, New Zealand

**Keywords:** Indigenous, Māori, public health services, COVID-19, case management, innovation, case-study

## Abstract

In Aotearoa/New Zealand (NZ), the Indigenous Māori population have been more severely impacted than non-Māori throughout the COVID-19 pandemic, and less well served by NZ’s COVID-19 response. This case-study describes an innovative Indigenous-led service delivery model, which was designed and implemented to improve the case and contact management of Māori with COVID-19 in Auckland. We outline the context in which the conventional public health case and contact management was failing Māori and the factors which enabled Indigenous innovation and leadership. We describe the details of the model and how the approach fundamentally differed to the conventional approach to care. Qualitative and quantitative data on impact of the model are shared, along with the key barriers and enablers in the implementation of the model. The Māori Regional Coordination Hub (MRCH) model offers a valuable alternative to the conventional public health case and contact management approach, and this case study highlights lessons which may be applicable to improving the design and delivery of public health services to other Indigenous and marginalized groups.

## 1 Introduction

Like many Indigenous groups worldwide (1–3), Māori in Aotearoa/New Zealand (NZ; representing 17% of the total population of 5.1 million) (4) have been more severely impacted than non-Māori from COVID-19. The age-standardized Māori hospitalization rate for COVID-19 is 2.3 times higher than people of “NZ European or Other” ethnicity (5). Māori are also 2.0 times more likely to die from COVID-19 (5), and Māori aged under 60 years are 3.7 times more likely to die from COVID-19, than people of “NZ European or Other” ethnicity (6).

Multiple social and health inequities place Māori at higher risk of COVID-19 transmission and more severe health consequences (7). Māori have on average the poorest health status of any ethnic group in NZ (8–10) yet Māori receive less access to, and poorer care throughout, the full spectrum of health care services from preventative to tertiary care (9, 11). Māori experience a higher burden of socioeconomic deprivation (9), household overcrowding, and higher rates of multiple co-morbidities at younger ages than non-Māori. However, only about a quarter of the higher age-adjusted COVID-19 mortality risk for Māori is explained by socioeconomic deprivation. After adjusting for factors such as sex, age, ethnicity, hospital-identified comorbidity and vaccination status, Māori still have 2.0 times the COVID-19 mortality of “NZ Europeans or Others” (6).

Health equity for Māori is a legislated responsibility of the government health system (12, 13). This focus reflects Te Tiriti o Waitangi, NZ’s foundational document which provides constitutional and legal obligations for the government to ensure equity for Māori. These Indigenous rights are also enshrined in the UN Declaration on the Rights of Indigenous Peoples (14). Despite NZ achieving lower mortality from COVID-19 than many countries, there is clear evidence that the public health response was less effective for Māori than non-Māori. An urgent inquiry by the government’s Waitangi Tribunal^1^ in 2021, found several aspects of the response disadvantaged Māori (15) and constituted significant breaches of Te Tiriti o Waitangi. This included the vaccination strategy, where the government rejected advice from its own officials to adopt lower age eligibility for Māori, and an overly rapid withdrawal of the government’s key COVID-19 protection measures before agreed vaccination coverage were met (15). Lower vaccination rates for Māori, and a rapid withdrawal of public health protections, exacerbated the disproportionate risk of COVID-19 for Māori, particularly during the Delta wave in late 2021, when NZ first experienced widespread community transmission.

This case-study describes development of the Māori Regional Coordination Hub for COVID-19 (MRCH) in the greater Auckland region. This innovative Indigenous-led service model was designed to improve case and contact management of Māori with COVID-19 in late 2021. This paper outlines the context in which the conventional public health case management was failing for Māori, and the factors which enabled Indigenous leadership. We describe details of the model, and how it fundamentally differed to the conventional approach. Qualitative and quantitative data on impact of the model are shared, along with key barriers and enablers we faced.

This account is positioned within a Kaupapa Māori research framework that: includes Indigenous Māori leadership and control of the contextual and data analysis, avoids victim-blame or cultural deficit positioning, privileges Māori/Indigenous experiences as participants within the processes that are being described and aims to support transformational change for health services to better meet Māori needs and aspirations (16).

We present this model as an Indigenous alternative to the conventional public health case and contact management approach, and highlight lessons which may be applicable to improving the design and delivery of public health services for other Indigenous and marginalized groups.

## 2 Evolution of COVID-19 response for Māori in Auckland

Indigenous-led innovations were rapidly implemented in Auckland, NZ’s largest city with a population of 1.65 million, shortly after the COVID-19 Delta wave commenced in mid-August 2021. Until this time, NZ’s elimination strategy for COVID-19 had successfully prevented widespread community transmission of the virus. By 1st August 2021, NZ had only seen 26 deaths from COVID-19 and just over 2,500 cases in total (17), most of which were detected and quarantined at the border. The arrival of the Delta strain led to a community outbreak in Auckland which was quickly outpacing the capacity of public health measures. This occurred at a time when most Māori were unprotected by vaccination, as NZ’s staged roll-out of COVID-19 vaccination to the general adult population only commenced on 28th July 2021, starting with people 60–64 years of age (18). Vaccination proceeded quickly, but by the peak of NZ’s Delta wave in November 2021 (19), only 77% of eligible Māori had received their first vaccination dose, compared to 94% of non-Māori, non-Pacific people (18).

Government public health measures in place at the time included region-wide restrictions on public movement except for essential purposes, mandatory face-masking in indoor public settings, mandatory home isolation for close contacts, and 14 day quarantine in government facilities for all positive COVID-19 cases (20). Laboratories automatically notified public health services of all positive COVID-19 results, and public health services then contacted each case by telephone to undertake contact tracing and case and contact management. The greater Auckland region was made up of three district health boards (DHBs), which jointly established a regional emergency structure, the Northern Region Health Coordination Centre (NRHCC), to coordinate the pandemic health response regionally. The region was served by a single public health service—the Auckland Regional Public Health Service (ARPHS).

Earlier in the pandemic in May 2020, the NRHCC Māori health team leads worked with ARPHS to improve the public health management of Māori COVID-19 cases, contacts and whānau (families). Initially this work focused on helping the (mostly non-Māori) public health professionals think through the questions and issues that might be relevant to ensure effective and efficient case and contact management for Māori. A Māori COVID case review in August 2020 produced further improvement recommendations for ARPHS and NRHCC to implement.

However, with the arrival of the Delta wave, weaknesses in the effectiveness of the public health case and contact management approach for Māori became more apparent. COVID-19 PCR testing was provided free of charge at a range of mass community testing centers, and existing health facilities. Once positive COVID-19 cases were notified to ARPHS, public health staff phoned the person to let them know their positive result and conducted an interview. This interview sought to identify high-risk events/settings and close contacts and asked about the household’s urgent welfare requirements for isolation. This interview followed a standardized script, commencing with a lengthy privacy disclosure statement, and was administered by mostly non-Māori staff. ARPHS would then arrange for the case to be transferred to quarantine (initially all cases were placed at government facilities, with a shift to most cases isolating at home as caseload increased), which was managed by another service. ARPHS had a team of Māori staff who were not authorized as contact tracers but were typically brought in to repair relationships when initial contact with Māori cases deteriorated. To be effective, this public health approach depended upon:

- Equitable access to COVID-19 health information and testing.
- Rapid processing of COVID-19 tests.
- Sufficient ARPHS staff to contact cases in a timely manner.
- Cases being contactable by phone.
- Culturally safe staff who could build rapport and trust over the phone.
- An efficient system to transfer cases to a safe place of isolation.
- Adequate welfare and clinical support to meet households’ needs while in isolation.

Barriers and delays existed for Māori at each of these steps, and weaknesses became more apparent as the caseload increased. Backlogs at all stages of the pathway meant it was sometimes several days before cases were informed they had COVID-19, with further delays for transfer to quarantine or receipt of urgent food/welfare support. In the Delta wave, approximately 50% of the COVID-19 cases were Māori and the outbreak became concentrated in some of the most socially disadvantaged Māori groups, including Māori previously poorly engaged with by health and other agencies. Where cases had telephones, they were often suspicious of unsolicited calls and disengaged if the initial contact was inadequate. These whānau were often in precarious health and social situations and not well placed to isolate safely, either at home or in quarantine facilities.

Given these challenges, ARPHS engaged senior external Māori public health physicians to provide support. This led to ARPHS sharing management of some complex Māori cases with Māori community health providers, with oversight from ARPHS. However, these community providers were not set up to manage all aspects of public health management and this hybrid approach failed to achieve the seamless, whānau-centered approach that was needed. The Māori expertise encouraged ARPHS to set up a Māori mobile team, Pae Ora Mobile (POM) in September 2021, to better respond to ‘hard-to-reach’ Māori whānau. This shift essentially flipped the traditional case interviewing approach, by taking a ‘whakawhanaungatanga (building relationships) and manaaki (caring for people) first’ approach to the engagement. In contrast to the traditional approach which sought to first elicit critical information about the case’s movements and contacts, culturally appropriate POM staff engaged in relationship building and took food parcels to cases’ homes as a first contact, ensured basic needs were met and established a level of trust, before seeking to elicit information relevant for contact tracing. POM staff were able to connect whānau with other trusted providers for follow-up care, or where this was not possible, become the default provider for COVID-19 testing (which was important to remove barriers for whānau who had difficulties getting to a testing center), clinical referral and isolation needs. In partnership with local mana whenua (Māori who have historic and territorial rights over the land) a second POM team with Ngāti Whātua ki Ōrakei was set up in November 2021.

As case numbers further increased during the Delta wave, delays across the COVID-19 pathway intensified. Once Auckland COVID-19 case numbers hit over 100 cases per day, the previous approach to case management became unsustainable and changes were required. The response to date had prioritized the assessment of public health risk, adding further delays to welfare and clinical needs being identified and met. The experiences of the POM team emphasized that caring for Māori with COVID-19 required simultaneously responding to the clinical, welfare and public health needs, in a culturally safe way, with minimal handovers. This point was further highlighted in late 2021 with a number of deaths (not necessarily from COVID-19) at home of people who were in COVID-19 home isolation, and increasing problems with government quarantine facilities being inappropriate settings for people with complex mental health, addiction and social needs. This prompted a call from Māori health experts advising ARPHS to shift from prioritizing case and contact tracing toward an overall focus on ‘saving Māori lives’.

## 3 Establishment of the Māori regional coordination hub

In response to these pressures, and to ensure an effective response for Māori with COVID-19, the Chief Executives of the DHBs who chaired the NRHCC, accepted a proposal from Māori experts to establish a Māori Regional Coordination Hub (MRCH) in December 2021. The MRCH model was proposed by the Māori leadership of the POM team and external Māori public health experts, and representatives from Ngāti Whātua ki Ōrakei. A project establishment team was set up within NRHCC, reporting directly to the General Manager, Māori Health for Auckland and Waitematā DHBs. An initial Māori workforce was seconded from ARPHS, Māori providers and externally recruited. To ensure Māori leadership over the model, MRCH reported directly to the NRHCC Director of Māori and was supported by a governance group consisting of senior Māori health and public health experts. The MRCH was operational within 3 weeks of inception.

The MRCH model consisted of a centralized Māori-led hub, with the following key components:

- A single place where all Māori COVID-19 cases in the Auckland region were notified.
- Culturally-safe staff undertook combined assessment of public health, clinical and welfare needs, using an evidence-informed (21) Māori screening assessment.
- An electronic triage system, scoring dimensions of both clinical and social risk, to prioritize capacity so the most high-risk people were assessed first.
- End-to-end visibility of cases throughout the COVID-19 pathway to ensure people were safely managed.
- Linked with a network of Māori community providers to deliver the most appropriate, holistic care for whānau (range of solutions for different needs).
- Minimized the number of providers/transfers involved in care, and maintained oversight to ensure no Māori fell through the cracks.
- Māori-led solution compliant with government’s obligations under Te Tiriti o Waitangi, co-designed with Māori and mana whenua.
- Māori governance, including Māori public health and health service delivery experts.

## 4 What MRCH did

**Figure 1:**
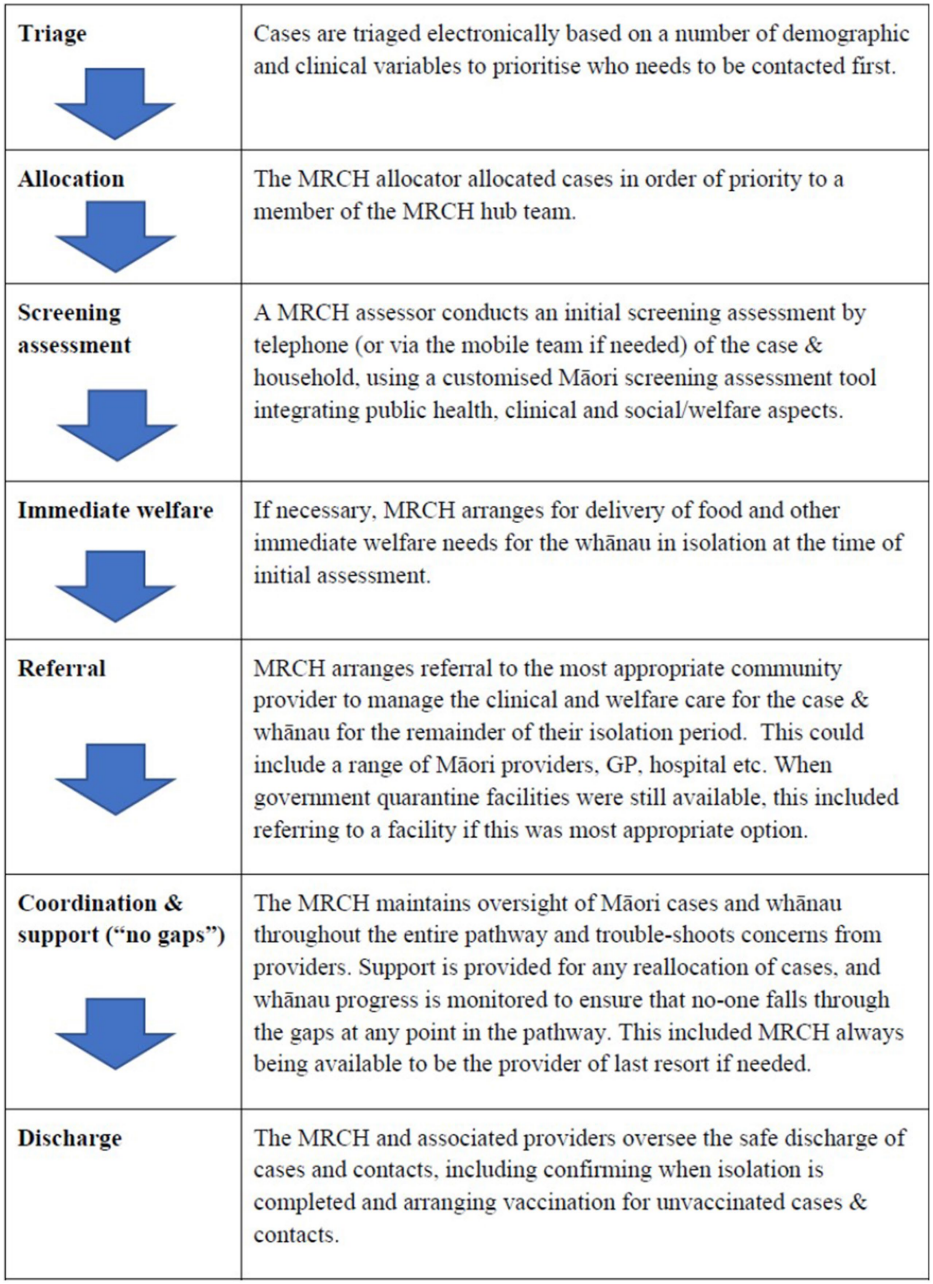
Pathway of core functions of MRCH.

MRCH handled the receipt, desktop triage, initial assessment and referral of all notified Māori COVID-19 cases in the Auckland Region (Figure 1). Cases underwent automated desktop triage based on:

1. Clinical risk—based on a custom-made score developed by NRHCC to identify risk factors, available in clinical databases, associated with higher risk of COVID-19 hospitalization (e.g., chronic kidney disease, immunocompromise, polypharmacy, obesity, cancer, lung disease, unvaccinated, diabetes, heart disease, age).
2. Social risk/indicators of disengagement—building upon a set of variables developed by the POM team, to identify Māori with high levels of social need (e.g., NZ Deprivation score (22), not enrolled with primary care).

Cases were classified as either high, medium or low risk on each of these scores, and considered high risk if they received a “high” score on either scale. Triaged cases then received a culturally appropriate phone contact from the MRCH team. During the Delta wave, the MRCH team sought to contact all Māori cases (high, medium and low risk) by telephone within 24 h of notification, with mobile outreach from team if needed to establish contact.

A key difference between the MRCH model and the mainstream approach, was that MRCH simultaneously assessed clinical, public health and social needs. This was important, given the high levels of poverty, comorbidity, household overcrowding and unmet social need among Māori, which made mandatory home isolation especially precarious. The most life-threatening needs for whānau were often not specific to COVID-19, but related to pre-existing chronic conditions, mental health issues or addictions. Being unable to leave home was especially dangerous for whānau without food stockpiles at home, or reliant on prepaid electricity, or without mobile phone credit to call for help if needed or experiencing conflict in the home. Interviewers used a screening assessment tool specifically developed by MRCH, based on a Māori approach to clinical interviewing (21), which prioritizes establishing relationships and providing welfare, before delving into more clinical questioning. Clinical oversight of cases managed by MRCH was facilitated via the employment of MRCH-specific clinicians (including general practitioners, nurse practitioners and hospital specialists). There was also some mobile capacity within the MRCH team, to provide emergency welfare directly to whānau unable to be reached by an alternative Māori provider.

The MRCH response shifted as the nature of the pandemic changed. Just before the COVID-19 Omicron wave arrived in January 2022, MRCH leadership proactively developed a plan to respond to whānau needs in the changing situation. This included identifying more proactive activities to prepare Māori, including Māori communications, and distributing basic health kits, including disposable masks, Rapid Antigen Tests (RATs) and symptomatic treatments. Unfortunately, this MRCH advice was not utilized, and resources were not made available for these activities to occur. The MRCH leadership team also predicted that the team’s human resource would not be able to match the expected demand in an Omicron wave. Workforce needs were escalated to NRHCC leadership, and MRCH leadership prioritized team operations so that on a given day at least those Māori cases designated as medium or high risk would receive a phone contact.

In response to pressures during the height of the Omicron wave, which saw a peak of 1,645 Māori cases notified to MRCH per day, further adjustments had to be made to ensure the team provided the best service with available capacity:

1. The screening assessment tool was shortened to a “power screening assessment” tool—enabling staff to complete more assessments within a given day while still assessing critical factors related to clinical, welfare and public health needs. The approach to triage scoring remained unchanged.
2. On days when the numbers of high/medium risk Māori cases were too great for MRCH to contact within the day, a switch to “safety check” calls was made—deferring the screening assessment to ensure that all new cases were contacted to establish that they were safe and there were no life-threatening health or social needs requiring immediate action.

MRCH worked directly with a network of 12 Māori community providers to ensure that Māori with COVID-19 had urgent clinical, welfare and public health needs met. All providers offered welfare/social support and five also offered clinical care.

## 5 Assessment of MRCH impact

### 5.1 Quantitative data

**Figure 2:**
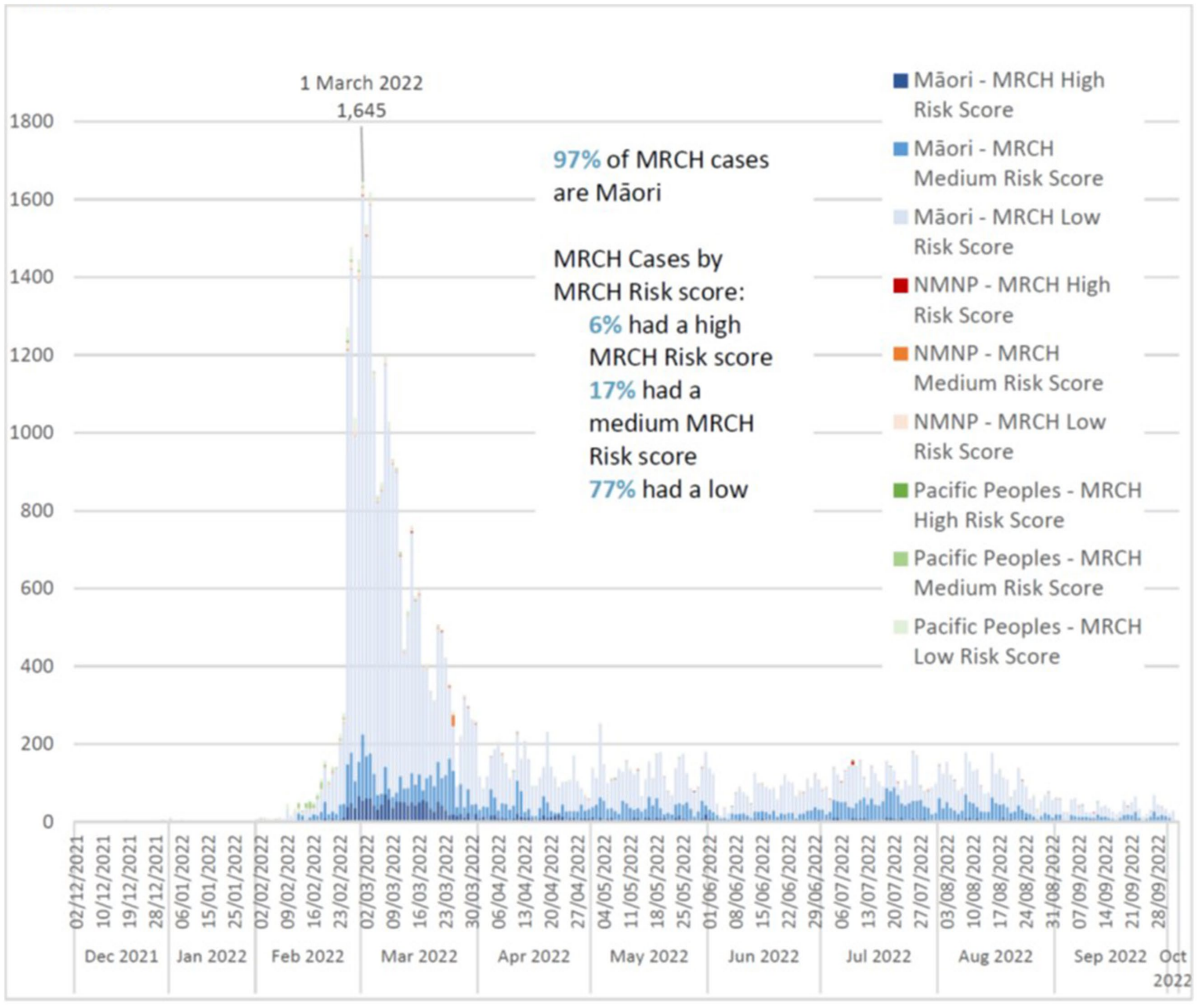
Total daily MRCH cases, December 2021–October 2022, by risk score and ethnicity. These case numbers will under-report the true number of cases referred to and assessed by MRCH, as any cases transferred by MRCH to other facilities (e.g., hospital) will be counted under that final facility, not the point of initial assessment. NMNP = people of non-Mäori and non-Pacific ethnicity (mostly NZ Europeans).

Between December 2021 and October 2022, MRCH managed over 46,000 cases, 23% of whom were high or medium risk, and made approximately 8,000 referrals to Māori providers for wrap-around clinical and social care. Over 97% of MRCH cases identified as Māori, and 7.8% were not enrolled with primary care. The daily case numbers varied significantly, as shown in Figure 2, and MRCH managed a peak of 1,645 cases on a single day at the beginning of March 2022. Not all cases were Māori, as a key principle of MRCH was to care for the entire household, and some Māori whānau have members of other ethnicities. Data are not available for the entire period, but between June–October 2022, over 87% of MRCH high risk cases and 89% of medium risk cases received a phone assessment within 24 h of notification. While approved for a quota of 41 full time equivalent (FTE) staff, MRCH functioned with a maximum FTE capacity of 21.2 FTE. The Delta outbreak was producing less than 10 cases per day by late January 2022.

**Figure 3:**
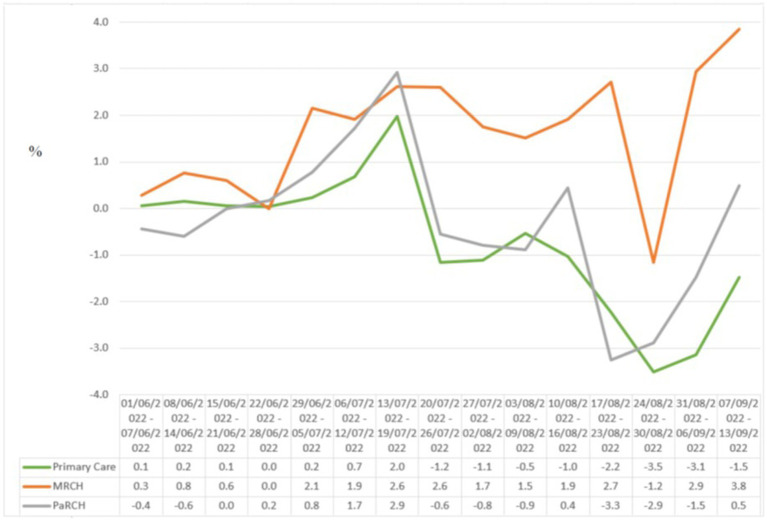
Difference between percentage of clients eligible, and percentage of clients who were dispensed COVID-19 anti-viral medications, for MRCH and other Auckland regional providers. Notes: A positive % on the y-axis means that the amount of anti-virals dispensed is above the percentage eligible (based upon PHARMAC’s single eligibility criteria), and a negative value indicates a dispensing rate lower than percentage of the population eligible, Ideally, the line in this graph would not drop below zero, indicating that dispensing levels, at a minimum, match the levels of population need. PaRCH, Pacific Regional Coordination hub, which is a COVID-19 hub for Pacific Peoples, inspired by the MRCH approach.

MRCH played a key role in providing access to COVID-19 anti-viral medications. Across Northern NZ, MRCH consistently achieved higher rates of dispensing than conventional services. Figure 3 shows the percentage of eligible people who received COVID-19 anti-viral medication, through each of the NRHCC COVID-19 service arms. By comparing difference between the percentage eligible and percentage dispensed across time, it can be seen that MRCH performed better than the other service arms—consistently meeting the ‘minimum’ criteria with no downwards trend. In terms of workload, a high percentage of the MRCH patient cohort needed anti-viral medication—of all patients notified to MRCH, around 10% under the initial eligibility criteria, and 20–30% under the current eligibility criteria, needed anti-viral medication.

### 5.2 Qualitative data

In addition to COVID-19 specific needs, MRCH found that Māori households frequently presented with unmet needs, including for:

- Food, money, and utilities (electricity, phone and internet),
- Basic supplies for cooking and warmth (functional stove, blankets, clothing),
- Safety (violence in home),
- Housing/homelessness
- Baby and child requirements (nappies, formula, education/entertainment needs),
- Personal care needs (e.g., incontinence pads and menstrual supplies),
- Animal welfare (e.g., pet food, emergency care when owners moved to another facility)
- Inadequately managed comorbidities (including mental health, addictions, diabetes, cancer and other serious long-term conditions)
- Essential medications (for pre-existing or long-term conditions),
- Essential medical devices (e.g., asthma inhalers),
- Healthcare (e.g., no GP, awaiting hospital appointments or surgical intervention, recent discharge from hospital requiring post-discharge care, acute non-COVID issues requiring hospitalization).
- Preventative health needs (e.g., vaccination, pregnancy).

As a service funded for COVID-19, MRCH staff assessed and met the COVID-19 needs of cases and their households (including monitoring for deterioration, arranging anti-virals, oximeters, PCR/RAT testing, vaccination), as well as addressing unmet health and social needs to keep Māori whānau safe and alive while isolating at home. MRCH, and the network of providers, assessed and managed the needs of the entire household. This was essential given the high proportion of Māori with precarious health and social situations, for whom the requirement to isolate at home for 7–14 days posed significant additional risk to their wellbeing. MRCH also provided additional community activities (e.g., Māori community vaccination days, provision of RATS, engagement with ‘hard to reach’ whānau and their respected leaders).

Through the entry point of COVID-19, MRCH was able to not only help address whanau’s critical immediate needs, but also help connect and advocate for Māori with other health and social providers. This included helping whānau enroll with permanent primary care providers, and arranging acute specialist hospital review for non-COVID-19 problems. MRCH encountered many Māori whānau needing health support from a range of services, who frequently found the experience of trying to access care disempowering, confusing and racist. Consequently, many whānau were falling through the cracks and MRCH helped navigate the complex matrix of services. This highlighted that, beyond COVID-19, there is an ongoing need for integrated, wrap-around culturally appropriate services to help Māori successfully access healthcare services they require.

## 6 Discussion

MRCH provided a unique contribution to the COVID-19 response for Māori in the Auckland Region. It was a Māori-led service, which sought to ensure all Māori with COVID-19 in Auckland had their needs (regardless of the type of need) assessed and met, in a culturally appropriate and timely way, focusing on those most at risk first. There have been several key lessons and issues arising from the establishment of MRCH, which are useful to highlight.

- **A “clip-on” approach to address weaknesses of conventional service vs. using Indigenous expertise to design an appropriate model from scratch**
  We wish to highlight a fundamental difference in approach to addressing unmet Māori needs between the MRCH model and the conventional public health response. When failures for Māori were observed at ARPHS, the approach was to bring in extra Māori staff as ‘clip-on’ cultural navigators to help deliver the conventional service/policy to Māori. Public health practice in NZ is grounded strongly in British colonial understandings and approaches. A failure to acknowledge this in-built bias meant that the fundamental approach to case and contact management was never up for debate or question—the focus was on how to achieve better Māori compliance with this conventional approach. This service-focused approach is fundamentally different from the POM and MRCH approach, led and designed by Māori, which started from a focus on Māori and designed the service to best meet Māori needs. Māori community (rather than just health professional) expertise was valued and incorporated into the MRCH model, including training non-clinical Māori staff as contact tracers (rather than using this expertise as cultural support for non-Māori staff).
- **Friction and resistance were encountered to power-sharing with Māori**
  Friction arose during the establishment of POM and MRCH, between the Māori leadership and the conventional organizations. This tension related to different worldviews, and different understandings of Indigenous leadership, partnership and power-sharing. While initially inviting in Indigenous expertise and being supportive of service changes, ARPHS continued to try and maintain control over the POM approach. There was a fundamental tension between this “governing of Indigenous governance” and enabling true power-sharing and Indigenous leadership. This was also expressed through ARPHS applying a higher degree of scrutiny to Māori-led solutions such as POM, than to the performance of the conventional service, including using a NZ European lens to evaluate Indigenous models. These tensions highlight why MRCH needed to be established independent of ARPHS. Unresolved tensions also compromised the ability for MRCH expertise to be viewed as partners in co-designing and improving (rather than just operationalizing) the broader public health response for Māori in the Auckland Region.
- **Crisis offered opportunities and challenges for Indigenous-led innovation**
  Our experience is that government health agencies share power with Māori reluctantly and only when facing significant risk of failure. In the 2021 Delta wave, conventional public health approaches were losing control of COVID-19 spreading among Māori communities, and this crisis created a willingness to support Indigenous-led solutions that is not normally present. However, innovating in a crisis also presented its own challenges. The rapidly evolving pandemic meant that service models were never static, and staff had to adapt rapidly. This rapid development meant that approaches had little time for testing or evaluation, and operational capacity was frequently overwhelmed. Achieving adequate staffing levels was an ongoing challenge. Reasons for this included shortages of staff with necessary attributes and recruitment delays. There were also contractual barriers, including short-term contracts and remuneration that was not competitive, especially for staff with specialized Māori expertise. MRCH staff retention was also affected by a lack of investment in staff professional development and capacity building, differing expectations of how a Māori service should operate, and continued uncertainty about the longevity of the model and thus security of employment. This highlights that providing certainty and staff development are still crucially important even in a crisis.
- **Data systems unable to answer key equity questions for Māori**
  Information systems for COVID-19 case management were being built and refined as the pandemic evolved. Software used to capture COVID-19 information was not designed to monitor performance on equity, and service-level reporting focused heavily on activity measures (e.g., numbers of cases under the care of each service) but did not enable any assessment of completeness, equity or quality of these services for Māori. This limits the degree to which we can answer key questions about MRCH, and limits the government’s ability to monitor whether the COVID-19 response was adequate to meet Māori needs and its obligations under Te Tiriti. Key questions we would ideally, but are unable to, answer include:
  - Did all Māori cases in Auckland actually get referred to MRCH?
  - Was MRCH able to contact all high and medium risk Māori cases, at all and within the targeted 24 h timeframe (this data is only available from June 2022)?
  - Was the triage system effective at successfully identifying those Māori cases most at risk (e.g., how many Māori triaged as low risk died, needed hospital or had urgent social needs?) The decision by MRCH to only contact high or high/medium risk cases was purely based on capacity and there is a need to monitor whether this was a safe level of service for Māori.
  - Was there sufficient provider capacity to manage referrals from MRCH, and were these responded to in a timely way?
  - How many Māori under the care of MRCH died and was there anything that could have been done to improve the care in those cases?
- **Government policy changes impacted the ability for MRCH to keep Māori safe**
  The ability of MRCH to keep Māori safe was heavily influenced by government decisions about the broader pandemic response. In response to the Omicron wave in early 2022, the government made policy changes which shifted toward increased personal self-reliance. This included a shift to self-testing and self-reporting, requiring cases to complete a lengthy online form to notify their positive COVID-19 result. This introduced additional access barriers for Māori and may have made it less likely that all Māori cases with COVID-19 were tested or notified (a prerequisite for MRCH engagement). In early 2022, there was also a policy change in how welfare was provided for COVID-19 cases, whereby the Ministry of Social Development (MSD) received and managed all welfare referrals directly, bypassing MRCH. This created serious concerns for whānau, many of whom reported to MRCH staff that they had significant distrust/poor experiences with MSD. At the height of the Omicron wave, MSD took 5–6 days to process welfare referrals, while at peak caseload MRCH providers took less than 48 h. Māori providers learned an extensive amount through providing welfare support to isolating whānau throughout the pandemic, and this policy change ignored those important learnings.
- **Difficulty in being recognized as a unique model, and erosion of core components over time**
  The MRCH model was distinctly different to the other community care models for COVID-19 provided by NRHCC. To protect the Indigenous governance of the model, MRCH had independent lines of accountability directly to the NRHCC Māori lead and did not report to the conventional organizational leadership for COVID-19 community care. This was important and appropriate for a Māori-led service, but we encountered misunderstanding of the MRCH service delivery model within the NRHCC itself, and with other partners including the Ministry of Health. MRCH service level data was often compared or reported alongside data for other populations groups, which were not comparable as the service provided was fundamentally different. It also led to contested ownership of the MRCH model in the retelling of NZ’s COVID-19 story. While mainstream agencies played an important role by enabling and supporting a Māori-led approach, the development and implementation of the MRCH model cannot be claimed by ARPHS, NRHCC or the Ministry of Health.

As the pandemic subsided, interest in supporting innovation dissipated. In addition to fulfilling a valuable role in keeping Māori in Auckland safe from COVID-19, the MRCH model offers a valuable alternative to the conventional public health approach. It is a Treaty-compliant approach which enables Māori to exercise sovereignty and self-determination over their public health response and care. This case-study raises an important question of how health systems can maintain and build upon innovation borne out of crises, especially approaches to reach Indigenous and marginalized groups, rather than reverting to an inequitable status quo.

## 7 Conclusion

MRCH was designed by Māori health leaders in Auckland in December 2021, in recognition of the inequitable COVID-19 burden, with the paramount objective of “saving Māori lives.” The model reached a highly vulnerable population, demonstrated by the high levels of clinical and social risk, and eligibility for anti-viral medication. This innovative Māori-led solution informed the development of the national triage tool for COVID-19 and service delivery approaches to provide better COVID-19 care for Māori across the country. This model required managerial courage and a willingness to share power with Māori to enable innovation. The MRCH model of care is not unique to COVID-19 and could be applied to a range of other clinical/social entry points, to improve the way government health and social services meet the needs of Māori. This case-study highlights lessons which may be applicable to improving the design and delivery of public health services to other Indigenous and marginalized groups.

## Data availability statement

The datasets presented in this article are not readily available because the datasets analyzed for this study are not available as is consistent with the Kaupapa Māori positioning of this paper. Requests to access the datasets should be directed to e.curtis@auckland.ac.nz.

## Author contributions

EC: Conceptualization, Formal analysis, Investigation, Methodology, Supervision, Writing – original draft, Writing – review & editing. BL: Investigation, Methodology, Project administration, Writing – original draft, Writing – review & editing, Conceptualization, Formal analysis. KL: Conceptualization, Methodology, Supervision, Writing – review & editing. AJ: Conceptualization, Data curation, Formal analysis, Funding acquisition, Project administration, Supervision, Writing – review & editing. NC: Conceptualization, Funding acquisition, Resources, Supervision, Writing – review & editing. RH: Conceptualization, Supervision, Writing – review & editing. KS: Conceptualization, Funding acquisition, Methodology, Supervision, Writing – review & editing. KT: Conceptualization, Data curation, Funding acquisition, Investigation, Methodology, Project administration, Writing – review & editing. PM: Project administration, Writing – review & editing. RT: Conceptualization, Data curation, Investigation, Methodology, Supervision, Writing – review & editing. SW: Conceptualization, Investigation, Project administration, Writing – review & editing. KE: Conceptualization, Data curation, Investigation, Methodology, Project administration, Writing – review & editing. RM: Conceptualization, Formal analysis, Funding acquisition, Investigation, Methodology, Writing – review & editing.

## Footnotes

^1^The Waitangi Tribunal is a standing commission of inquiry, which makes recommendations on claims brought by Māori relating to legislation, policies, actions or omissions of the Crown that are alleged to breach the promises made in the Treaty of Waitangi, New Zealand’s founding document.
